# The Quest for the Hippocampal Memory Engram: From Theories to Experimental Evidence

**DOI:** 10.3389/fnbeh.2020.632019

**Published:** 2021-01-15

**Authors:** Omid Miry, Jie Li, Lu Chen

**Affiliations:** ^1^Department of Neurosurgery, Stanford University School of Medicine, Stanford, CA, United States; ^2^Department of Psychiatry and Behavioral Sciences, Stanford University School of Medicine, Stanford, CA, United States

**Keywords:** engram, hippocampus, memory, learning, place

## Abstract

More than a century after Richard Semon's theoretical proposal of the memory engram, technological advancements have finally enabled experimental access to engram cells and their functional contents. In this review, we summarize theories and their experimental support regarding hippocampal memory engram formation and function. Specifically, we discuss recent advances in the engram field which help to reconcile two main theories for how the hippocampus supports memory formation: The Memory Indexing and Cognitive Map theories. We also highlight the latest evidence for engram allocation mechanisms through which memories can be linked or separately encoded. Finally, we identify unanswered questions for future investigations, through which a more comprehensive understanding of memory formation and retrieval may be achieved.

## Introduction

In order for learning to occur, an experience must result in enduring changes in anatomical connections and physiological processes within the brain. Rapid and efficient recall of this experience (e.g., an episodic memory) depends on these anatomic and physiologic changes. The sparse ensemble of neurons across multiple brain regions manifesting these learning-induced changes is called an *engram*. This concept that learning induces persistent alterations in a subset of neurons was first proposed by the German scholar and theorist Richard Semon, who defined the engram as “*.the enduring though primarily latent modifications in the irritable substance produced by a stimulus*.” (Semon and Simon, 1921). Based on his “*Law of Engraphy,”* Semon postulated that “*All simultaneous excitations.within our organisms form a connected simultaneous complex of excitations which, as such, acts engraphically, that is to say leaves behind it a connected and, to that extent, unified engram-complex”* (Semon et al., 1923). Today this “connected and unified engram-complex” is interchangeable with “memory trace” and is widely recognized as the substrate for episodic memories in the brain, an assembly of cells which are: (1) activated during experience, (2) undergo structural and functional modifications as a result, and (3) reactivated upon recall of the experience (Tonegawa et al., 2015).

The quest for the engram began more than a century ago. American psychologist and behaviorist Karl Lashley systematically tried and failed to find the engram by ablating cortical tissue at varying locations in the rat after maze-learning, and concluded that “*This can only mean that the retention of the habit is conditioned by the total amount of functional tissue in the cortex and not, primarily, by the inherent properties of the synapses themselves*” (Lashley, 1929). Existence of the engram remained a theory for most of the twentieth century, even as Donald Hebb's supportive doctrine that “neurons which fire together, wire together” (Hebb, 1949) was validated experimentally by characterization of long-lasting, activity-dependent changes in synaptic strength between co-active neurons (Bliss and Lomo, 1973; Collingridge et al., 1983; Malenka and Bear, 2004). Activity-induced changes in synaptic strength between co-active neurons, also known as *associative plasticity*, was extensively characterized in the ensuing decades, providing detailed insight into how the second criteria for the theoretical engram is established (Nicoll, 2017). Still, limited scientific tools and the sparse nature of the engram prolonged its elusiveness. It took the discovery that neurons express the proto-onco-gene *Fos* upon robust stimulation (Curran and Morgan, 1985), and that expression of *Fos* is a reliable indicator for plasticity-inducing activity in neurons *in vivo* (Morgan et al., 1987) for the prospect for access to the engram to emerge.

Indeed, after discovery of this genetic proxy for neural activity, expression of *Fos* (Radulovic et al., 1998), as well as other activity-induced Immediate-Early Genes (IEGs) including *Arc* (Guzowski et al., 1999) and Z*if/*268 (Hall et al., 2001) was co-opted to gain optical access to the engram after salient experiences ranging from exposure to a novel environment (Guzowski et al., 1999) to auditory fear conditioning (Hall et al., 2001). Using *post-hoc* immunohistochemistry or *in situ* hybridization to label the engram in brain sections, studies from the amygdala, hippocampus, and cortex have reliably visualized a sparse population of neurons that reactivated at a greater-than-chance level upon re-exposure to the environment previously experienced (Guzowski et al., 1999; Hall et al., 2001). To experimentally demonstrate the third criteria, that engram reactivation underlies memory recall, further scientific advances were needed to enable genetic “tagging” of engram cells based on IEG expression, and to allow permanent access to these cells for manipulating their accessibility during memory recall (Reijmers et al., 2007; Guenthner et al., 2013; Tayler et al., 2013; Sorensen et al., 2016). It was subsequently confirmed that ablating the amygdala fear memory engram by IEG-driven expression of the *diphtheria toxin* receptor in engram cells followed by administration of *diphtheria toxin* abolished conditioned fear response (freezing) in mice (Han et al., 2009). Conversely, when the engram was artificially activated remotely via expression of the light-gated excitatory channel *channelrhodopsin-2* in hippocampal engram cells, conditioned fear response was elicited without any external cues (e.g., fear-training context) (Liu et al., 2012).

With all three criteria for the theoretical engram validated experimentally, and with the genetic and biophysical tools to access and manipulate the engram, we find ourselves in a historic position to elucidate outstanding questions about Semon's theory. Specifically, *what* information is encoded in the engram, and *how* is the information encoded? In this review, with focused attention on the hippocampal engram and its role in episodic memories, we highlight the latest evidence that addresses these questions, and underscore new questions in the field. If the engram is analogous to nodes within a network that enables storage and retrieval of experience, characterization of the laws that govern its assembly, maintenance, and erasure will be essential toward understanding its computational significance and implications. We begin by contrasting two theories of how the hippocampus supports episodic memory, and how new discoveries in the engram field reconcile the theories.

## The Hippocampal Engram and Memory

### Indexing Experience

The hippocampus is known to be essential for formation of episodic memories (Scoville and Milner, 1957). Engram units within the hippocampal subregion CA1 have long been postulated to serve as “index cells”—cells which have integrated pre-processed multisensory information from the entorhinal cortex with a simultaneous, highly filtered representation of this information from within the hippocampal circuit. As a result of this integration, CA1 neurons undergo the pertinent synaptic modifications to provide rapid access to the content of an experience stored in higher cortical areas [“The Memory Index Theory” (Teyler and DiScenna, 1986; Teyler and Rudy, 2007; Tanaka and McHugh, 2018)]. According to this theory, the hippocampal engram holds no specific information and is agnostic to the contents of the experience, but rather binds the reactivation of downstream neocortical ensembles to retrieve episodic content: what, when, and where. The Memory Index Theory finds support in anatomical and circuit considerations (Swanson and Mogenson, 1981), well-characterized mechanisms of synaptic plasticity within these circuits (Bliss and Lomo, 1973; Collingridge et al., 1983; Malenka and Bear, 2004), and behavioral studies confirming conjunctive representations of experience (Fanselow, 1990; Rudy and O'Reilly, 1999, 2001; Cai et al., 2016). The most convincing evidence, however, comes from studies leveraging IEG expression to genetically tag and/or modulate neurons within the hippocampal engram. These studies demonstrate that silencing a subset of the hippocampal engram (loss-of-function studies) impairs fear memory recall (Denny et al., 2014; Matsuo, 2015) while artificially activating a subset of the engram (gain-of-function studies) in the absence of memory retrieval cues is sufficient to elicit the full fear memory response (Liu et al., 2012; Ghandour et al., 2019). That fear memory is only partially impaired by incomplete silencing of the entire engram but is elicited fully when activating only a subset of the engram, suggests engram ensemble activity contributes to the pattern completion role associated with CA1 and its input regions (Rolls, 1999). Further, by silencing CA1 engram neurons during memory retrieval, Tanaka and colleagues found compromised reactivation of cortical ensembles (Masamizu et al., 2014), confirming a direct link between the CA1 engram and cortical representations. Once an engram has formed, *in vivo* calcium imaging studies have revealed greater synchrony and higher repetitive activity in engram cells compared to non-engram cells, even in the absence of cue presentation (Ghandour et al., 2019; Zhou et al., 2020). Together, these data strongly support an indexing function for the hippocampal engram. A second theory, however, emphasizes that the hippocampal engram serves as more than just a “trace without content,” that it computes and contributes information, namely location and context, necessary to anchor the information as an experience.

### Mapping Experience

The prevailing current model holds that the CA1 engram for episodic memories is assembled based on the animal's current location within the cognitive representation of space [The Cognitive Map Theory (O'Keefe and Nadel, 1978)]. This theory is supported by the observation that most CA1 neurons fire in a sparse, spatially selective manner (O'Keefe and Dostrovsky, 1971), and importantly, the firing rate of these “place cells” is influenced by contextual variables such as the presence of local cues (i.e., tactual, visual, or olfactory cues in the arena) (Bostock et al., 1991; Shapiro et al., 1997; Leutgeb et al., 2004) or the allocentric relationship of the rodent to distal spatial cues (i.e., distance and orientation of cues) (Hetherington and Shapiro, 1997; Cressant et al., 2002; Leutgeb et al., 2004). Further, the behavioral state of the rodent, such as goal orientation in a task (Markus et al., 1995; Shapiro et al., 1997) or memory associated with an environment (Hollup et al., 2001; Moita et al., 2004) has been shown to elicit a shift in place cell firing rate, or “rate-remapping.” Thus, not only do hippocampal place cells provide a spatial code to map the environment (Wilson and McNaughton, 1993), their ability to undergo rate-remapping upon environmental influence provides an additional hierarchal code with links to episodic content. This location-specific modulation of firing rate is thought to drive synaptic plasticity processes that stabilize the maps and their associated episodic content so that they can remotely be recalled (Kentros et al., 1998; Ziv et al., 2013).

**Figure 1:**
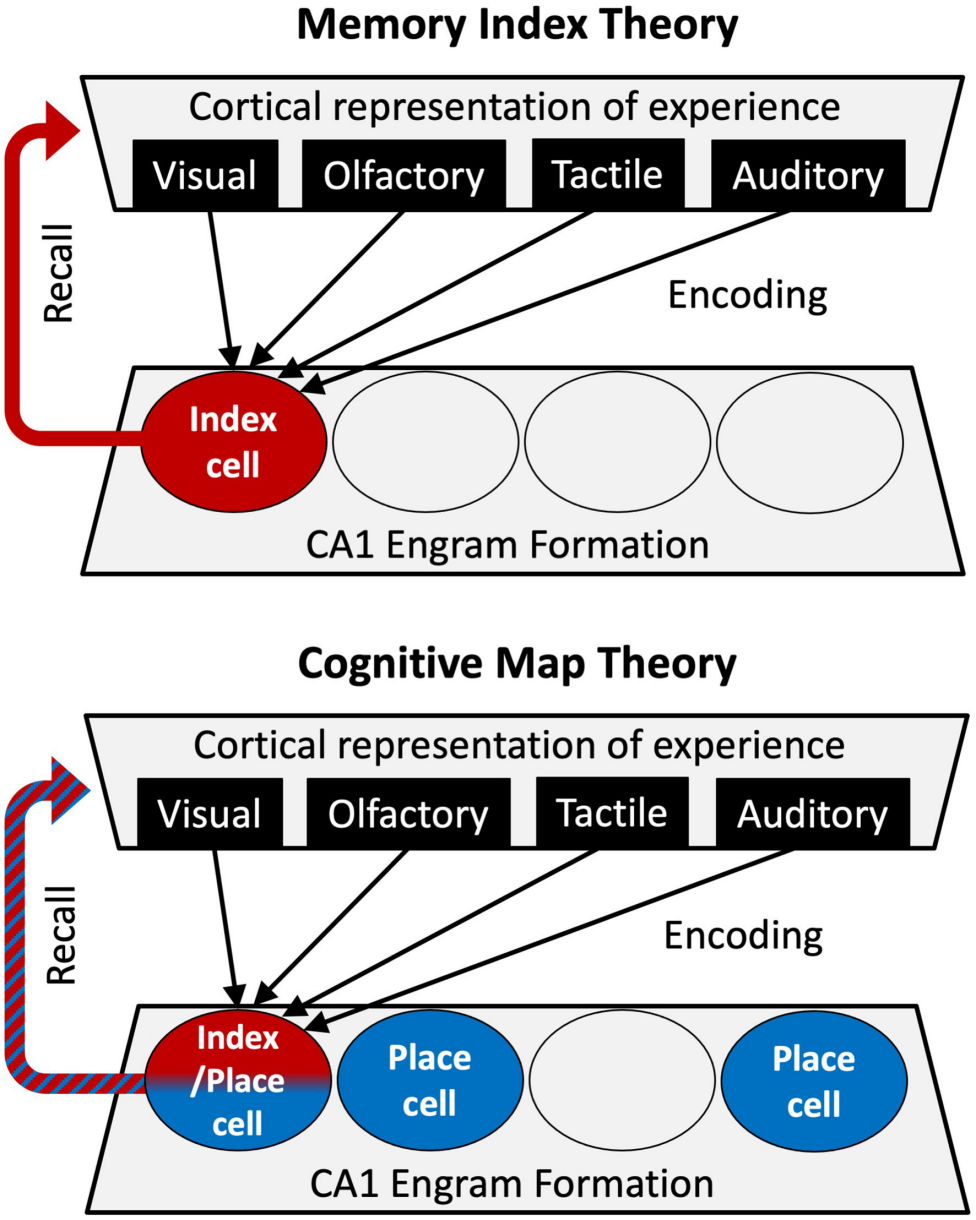
Comparing roles for hippocampal CA1 in the Memory Index theory **(Top)** and Cognitive Map theory **(Bottom)**. In the Memory Index theory, the CA1 engram cells (red index cell) merely integrate upstream representations of an experience to serve as an index for retrieval of the experience. In the Cognitive Map theory, the indexing of the experience (red cell) occurs on a substratum that encodes space (blue place cells), providing a context-based anchor for episodic memories.

With recognition that neurons within the hippocampus contribute indispensable information about an experience (location and context), the Cognitive Map interpretation has largely replaced the Memory Index Theory for the study of episodic memory (Figure 1). The Cognitive Map Theory, however, does not fully explain some of the most important aspects of episodic memory attributed to the CA1, such as temporal association (the binding of discontinuous elements of an experience in time) (Ahmed et al., 2020), and the formation of conjunctive representations, during which independent features of an experience are bound into a unitary representation (Rudy and O'Reilly, 1999). Furthermore, the location specific firing of place cells in a new environment is not necessarily a determinant of IEG expression (Miyashita et al., 2009; Tanaka et al., 2018). For example, not all CA1 engram cells tagged by IEG expression in a novel environment are associated with place fields in the arena, and the majority of place cells are not tagged in the engram (Tanaka et al., 2018). However, engram neurons that do form place fields in one environment are less likely than non-engram place cells to remap to a new location in a new environment. In other words, the engram place cells are more faithful to the environment they encoded at the time of being recruited to the engram. Together, these results suggest that the two theories are reconcilable, and are not entirely mutually exclusive. It is possible that some place cells, due to plasticity-inducing stimuli during an experience, lend themselves to become index cells. On the other hand, it is possible for neurons that do not develop a place field in an environment to become an index cell due to strong non-spatial input during experience. These two mechanisms can work synergistically to maintain contextual representations bound by episodic content. How can we differentiate between the contributions of the index function and the cognitive map in the formation and recollection of episodic memory? And how do they work together to support such a function?

### Approaches to Distinguish

Given that place field plasticity and engram cell plasticity utilize a common cast of effectors such as neurotransmitters, their receptors, and downstream biochemical signaling pathways, it is difficult to conduct experiments in which place cell activity is silenced while engrams are allowed to potentially assemble. Such an experiment would directly separate which features of an experience can be remembered without a cognitive map. For example, the *N*-methyl-D-aspartate (NMDA)-subtype glutamate receptor has been shown to be necessary for long-term maintenance of hippocampal place maps (McHugh et al., 1996; Kentros et al., 1998), but is already a well-established contributor to learning due to its “coincidence detector” nature at the synapse (Collingridge et al., 1983). Similarly, overlapping circuitry prevents selective silencing of spatial information inputs or episodic information inputs onto engram cells. For instance, lesioning the septal area can abolish place map stability (Leutgeb and Mizumori, 1999), but because this brain region also supports many other aspects of experience, inferences cannot be made on the relationship between space and memory.

Another major obstacle in studying the relationship between place cell physiology and memory is the reliance on active navigation in memory-dependent tasks. Rodents learn an environment by locomotion, physically exploring every aspect of an arena to experience it. Locomotion has strong influence on hippocampal physiology, from activation of oscillatory brain rhythms (Buzsaki et al., 2003) to engagement of speed-activated interneurons within CA1 (Gois and Tort, 2018), and these influences are likely to impose an additional organization on contextual representation. The potential significance of this altered organization is appreciable when considering that in primates, who visually scan an environment rather than actively explore it, spatial representation is influenced by hippocampal neurons that fire in a gaze-direction-specific manner (Rolls, 1999). Since there are few locomotion-associated influences in the primate's learning of the environment, it is likely that their engram assembly and organization may differ from the rodent.

Complicating interpretations further, most navigation-based memory tasks in rodents use linear track navigation, which allows precise control of the rodent's transition from one spatial location to the next. This experimental paradigm has been proven valuable for the study of navigation, from single neuron physiology to network analysis (Pfeiffer and Foster, 2013; Bittner et al., 2017), and has also been applied successfully to memory studies. For example, the replay of place cell sequences or correlation between place cell activity in the awake, resting animal (Foster and Wilson, 2006; Diba and Buzsaki, 2007; Karlsson and Frank, 2009) and during slow-wave sleep (Wilson and McNaughton, 1994; Skaggs and McNaughton, 1996; Lee and Wilson, 2002) has been interpreted to support consolidation of an experience into memory. One of the limitations of the design, however, is that this paradigm relies on reward learning to incentivize constant traversing across a linear track, repeatedly activating the same sequence of place cells in each direction as the rodent seeks reward (Dombeck et al., 2010; Rubin et al., 2015). Caution should be taken when interpreting results from these studies with the recognition that repeated traversal along a linear or circular track may impose a somewhat artificial organization on place map physiology, the rules of which may not be applicable to memory formation in general.

Future studies trying to reconcile the Cognitive Map theory with the Memory Index theory must overcome these technical limitations in order to elucidate how two different codes: one for place and one for binding features of the environment work together to support episodic memory. In the meantime, another key approach toward understanding engram function is to understand how the sparse constituents of the engram are recruited. What are the mechanisms mediating such sparse allocation? How is the allocation maintained? An understanding of these questions will usher a wide range of experimental strategies to better understand engram function.

## Memory Engram Allocation

### Neuronal Allocation

Neuronal allocation describes the phenomenon by which a specific population of neurons, but not others within the same network, are recruited to encode an experience as memory. Is this small subset of neurons chosen at random, or are they somehow predisposed to encode an experience? Studies with intracellular recordings in hippocampal CA1 neurons during novel environment exploration demonstrate that cells with relatively high excitability immediately before novel environment exploration are more likely to become place cells (Epsztein et al., 2011; Cohen et al., 2017) or learning-activated engram cells (Li et al., 2020), suggesting a certain degree of predetermination. Moreover, artificial excitation of a silent hippocampal CA1 neuron using current injection or photo-stimulation at a specific position in space transform it into a place cell encoding that position (Lee et al., 2012; Rickgauer et al., 2014). These early observations led to the excitability-based allocation hypothesis, which posits that intrinsic neuronal excitability influences a cell's chances of becoming part of an engram.

Insight on the mechanisms underlying this excitability-based allocation theory was provided by a study demonstrating that overexpression of the activity-induced transcription factor Ca2+/cAMP response element-binding protein (CREB) in the lateral amygdala before fear-learning enhances auditory fear memory (Josselyn et al., 2001). The causality between engram allocation and CREB expression was further established by manipulating CREB expression levels in individual neurons (thereby bi-directionally altering neuronal excitability) and examining their chance of being recruited into an engram ensemble (Han et al., 2007; Zhou et al., 2009). These evidences further validate the hypothesis that neurons with relatively higher intrinsic excitability win the allocation competition to become engram cells. Later studies expanded the excitability-based allocation hypothesis beyond the amygdala network to multiple brain regions, including dorsal CA1 region of hippocampus (Sekeres et al., 2012; Smith et al., 2016; Li et al., 2020), dentate gyrus (Park et al., 2016), prefrontal cortex (Matos et al., 2019), and the insular cortex (Kumar et al., 2012).

In addition to manipulation of CREB expression, the influence of neuronal excitability in memory allocation was also validated directly *in vivo*. For example, it was shown that memories of similar nature but belonging to two distinct experiences (e.g., two different spatial context) separated by a short time window are allocated to highly overlapping engram cells in the hippocampus, suggesting excitation from the first experience predisposes those cells to encode the second experience (Cai et al., 2016). Importantly, engram overlapping was proposed as the neural basis of memory linking–recall of one experience triggers recall of the second experience if their engrams have significant overlap (Cai et al., 2016). Additional evidence supporting neuronal excitability and memory linking has been reported from the lateral amygdala, where a fraction of neurons encoding one fear memory are found to also encode a second fear memory if the two events occur within hours (1.5 to 6 h) (Rashid et al., 2016). Intriguingly, in the amygdala, memories that are dissimilar in nature but similar in emotional valency (e.g., contextual fear conditioning vs. conditioned taste aversion memory) can be co-allocated to overlapping engram cells and can be co-retrieved (Yokose et al., 2017). The conclusions emerging from these studies strongly support the hypothesis that higher intrinsic excitability at the time of experience predisposes neurons to memory allocation.

### Synaptic Allocation

The neuronal allocation perspective gives high importance to neuronal properties in determining memory engram allocation—neurons that are more excitable at the time of learning become the bearers of the memory. Does the memory allocation process end once a population of neurons with higher excitability are designated as engram cells? A few lines of evidence suggest that additional processing and maintenance of the memory trace is required. First, the increase in neuronal excitability is only transient, and the enhanced excitability typically decays back to normal in a matter of hours to days (Moyer et al., 1996; Zhang and Linden, 2003). Memory, on the other hand, lasts for months to years (Disterhoft and Oh, 2006). How do engram cells maintain their fidelity to a particular memory after their excitability decays back to normal? Second, it has been demonstrated that different memory engrams may involve partially overlapping populations of neurons (i.e., individual neurons can participate in encoding multiple memories). If memory encoding is at neuronal excitability level only, then erasure of one particular memory (hence silencing of the engram ensemble for this memory) will cause significant distortion/impairment of other memory engrams. However, selective erasure of one memory without affecting other memories similar in nature has been experimentally demonstrated (Yokose et al., 2017; Abdou et al., 2018). Thus, beyond neuronal allocation, which treats individual neurons as memory storage units, there must exist another mechanism that further distributes memory traces to a sub-neuronal level. Given the established importance of synaptic plasticity in memory storage (Bliss and Collingridge, 1993; Morris and Frey, 1997), synaptic allocation is likely another major mechanism mediating memory engram allocation.

Synaptic allocation refers to the mechanisms that determine how synapses are involved in storing specific memories. Most neurons have hundreds to thousands of synapses (e.g., hippocampal pyramidal neurons), and previous studies suggest that an individual neuron may recruit 15–20% of all its synapses to encode a specific memory (Rumpel et al., 2005; Yang et al., 2009; Fu et al., 2012). How are these synapses recruited to participate in the memory trace? Here, we will focus our discussion of synapse recruitment on two key aspects: the spatial influence and the temporal (activity history) influence.

The spatial aspect concerns the spatial distribution of synapses involved in encoding a specific memory. Previous modeling has predicted that synapses carrying similar information tend to cluster together on local stretches of dendrites, and synchronous activation of these spatially adjacent synapses trigger local dendritic spiking and neuronal input-output transformation (Poirazi et al., 2003; Govindarajan et al., 2006; Ujfalussy and Makara, 2020). Consistent with predictions from modeling studies, *in vivo* imaging experiments demonstrate that learning-induced new spine formation occurs more often at neighboring synapses than at random (Takahashi et al., 2012; Winnubst et al., 2015). Specifically, the connectivity between hippocampal CA3 and CA1 neurons is found to be highly structured and clustered both at the neuronal and dendritic branch level (Druckmann et al., 2014). In addition, it has been shown that new spines formed during repeated motor learning tend to cluster at anatomically adjacent positions in the dendrite and the clustered spines are more stable than isolated ones (Xu et al., 2009; Fu et al., 2012). By contrast, new spine formation associated with distinct memories, although similar in nature (e.g., learning of two different motor tasks), do not cluster with each other (Fu et al., 2012), further supporting the notion that the process of synaptic allocation for new memories is well-orchestrated spatially. What drives spatially correlated formation of spines during memory encoding? One widely accepted explanation is that induction of LTP in one spine initiates complex biochemical signaling cascades in the local dendritic region. Some of these molecular events facilitate the cooperativity of LTP at nearby synapses, thus leading to the coordinated potentiation of neighboring synapses and promoting synaptic clustering (Harvey and Svoboda, 2007; De Roo et al., 2008; Murakoshi et al., 2011; Frank et al., 2018). Additionally, local resources of polyribosomes and smooth endoplasmic reticulum have been shown to promote local synaptic strengthening and structural plasticity, including clustered spine formation after synaptic plasticity induction (Chirillo et al., 2019).

In addition to the above-mentioned spatial constrains in synaptic memory allocation, the temporal aspect of memory allocation describes a timeframe during which modification of synaptic strength pertinent to engram encoding is heavily influenced by the activity history of the synapse. There are two temporally relevant events under consideration here. First, how do short-term changes in synaptic activity transform into long-lasting memory traces? Second, how does the recent activity history of a given synapse impact its participation in subsequent memory encoding?

The hallmark feature of a memory trace is its relatively long-lasting nature. To support an enduring memory, activation of gene transcription and protein synthesis must occur. However, these events occur in the cell nucleus (transcription) and mostly in the soma (protein synthesis). How do global events at cellular level transform to modification of specific synapses? The synaptic tagging and capture (STC) theory posits that plasticity-inducing activity produces a local tag at affected synapses, which allows plasticity-related proteins (PRPs) produced at the global level to be captured at specific synapses, thus converting a short-term synaptic change (e.g., early-LTP) into a long-lasting modification of synaptic strength (late-LTP) (Frey and Morris, 1997; Youngblood et al., 2010). STC theory additionally predicts that PRPs synthesized during consolidation of a strong memory can influence the consolidation of an unrelated weak memory trace involving the same neuron, thus stabilizing synaptic potentiation processes of the weak memory and promoting its storage rather than decay (Martin and Kosik, 2002). Due to the nature of selective potentiation, STC was considered to be a potential mechanism for memory allocation. Specifically, when an animal is acquiring a weak memory of a non-salient experience, and a neuron involved in the acquisition of this memory has recently participated in the acquisition of a strong memory of a salient experience, the synapses activated during weak-memory acquisition are expected to utilize the PRPs produced from the strong memory, increasing these synapses' chances of being allocated to a memory (Ballarini et al., 2009; Wang et al., 2010). Given that the synapses involved in acquisition of the weak memory are only likely to be potentiated in neurons where a recent strong memory is consolidated, the two memories are encoded in overlapped memory engrams and they are potentially linked (Kastellakis et al., 2016).

In addition to achieving enduring synapse-specific changes, STC theory also predicts another interesting feature of synaptic modification. Like scenes from a movie, daily life generates continuous streams of activity in neural networks throughout the brain. Only a portion of these activities leads to synaptic modification that are directly applied to new memory formation. Other activities, however, impact memory formation in a much more subtle manner. According to the STC theory, a short-term synaptic modification is not guaranteed to turn into a long-term one, and the availability of PRPs within the window of tag availability is the key determinant. Production of PRPs may be triggered by a strong initial synaptic activity that produces the short-term plasticity, or by unrelated strong heterosynaptic activation occurring within a short time window from the activity relevant to memory formation (Frey and Morris, 1997, 1998). Thus, the synaptic events leading to synaptic tagging and the PRPs production may be temporally dissociable, and other neural events, happening before or after memory encoding, could directly affect the persistence of memory by dictating the availability of PRPs. The term “metaplasticity” (the plasticity of plasticity), first coined by Bear and associates (Abraham and Bear, 1996), refers to a shift in the state of neurons or synapses that alters their ability to respond to a given stimuli. Metaplasticity integrates bouts of synaptic plasticity generated within minutes to days. One form of metaplasticity, Bienenstock-Cooper-Munro (BCM)-like threshold modification, has been engaged *in vivo* and shown to last up to 35 days in the dentate gyrus (Abraham et al., 2001). Exposure to an enriched environment (EE) has been widely used as a behavioral paradigm to induce metaplasticity. For example, a 14-day exposure to EE facilitates CA1 LTP induction for at least 6 subsequent weeks (Buschler and Manahan-Vaughan, 2012). EE experience has also been reported to enhance performance in various learning and memory tasks (Kuo et al., 2006; Kumar et al., 2012; Parsons and Davis, 2012; Avaliani et al., 2016). Thus, activity history of a synapse establishes the state of this synapse, dictating its modification in subsequent learning. In the context of memory encoding, metaplasticity theory predicts that whether and how a subset of synapses of a neuron participate in certain recent experience will impact how these synapses may be involved in encoding future memory engrams.

**Figure 2:**
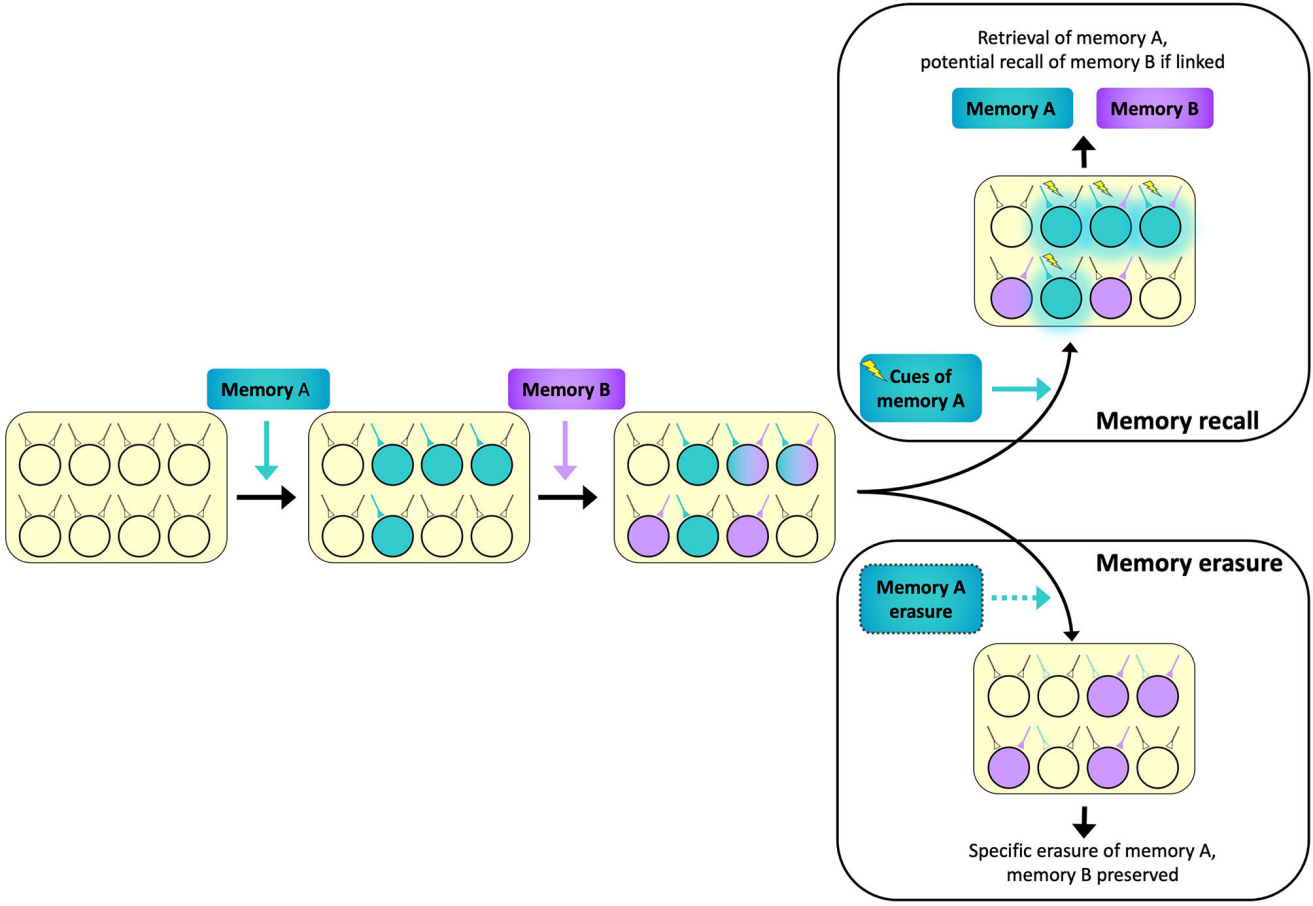
A schematic diagram illustrating two memory engrams allocated to overlapping neuronal ensembles to maximize network capacity for memory storage. The ensemble encoding Memory A (teal) partially overlaps with the ensemble encoding Memory B (purple). Activating the synaptic inputs which elicit recall of Memory A (lightning bolts) can potentially trigger recall of Memory B (teal glow) if the two memories are linked via substantial overlap in ensembles (Memory recall inset, top right). However, because Memory B maintains its unique set of inputs, erasure of Memory A will not interfere with Memory B (Memory erasure inset, bottom right).

We have summarized two potential factors determining memory engram allocation: neuronal allocation and synaptic allocation. Although the two mechanisms are different and are traditionally investigated separately, these two aspects of memory encoding potentially work synergistically: once a neuron is chosen to participate in encoding a specific memory, enduring synaptic changes will be induced at appropriate synapses (Martin et al., 2000). These changes may be long-lasting at the time scale of days to months, even years, thus transforming a transient increase in neuronal excitability to an enduring memory trace stored at specific synapses. Furthermore, at the circuit level, neuronal allocation and synaptic allocation mechanisms integrate to prevent memory interference during encoding (Varela et al., 2016; Titley et al., 2017). In other words, if related memories are linked in overlapping neurons through neuronal allocation, then synaptic allocation enhances the capacity of the network by providing specificity (Figure 2). This hypothesis is supported by evidence from behavioral studies: two associative memories learned within a time period of a few hours are known to be allocated to overlapped populations of neurons in both the hippocampus (Cai et al., 2016) and amygdala (Rashid et al., 2016). Silencing the overlapping engram cells between two memories interrupts the ability of one memory to trigger the recall of the other, but individual memories remained intact and could be elicited independently (Yokose et al., 2017). Furthermore, artificially potentiating or depressing the plasticity of synapses associated with one memory using optogenetic methods affects the recall of that specific memory while leaving the other memory uninterrupted, even though the two memories share overlapping engrams (Abdou et al., 2018). Taken together, these experiments further support the notion that neuronal allocation underlies memory linking while synapse-specific plasticity ensures memory fidelity and specificity.

## Summary

In a relatively short period of time, much progress has been made toward understanding how engrams are formed and how they function. We have reviewed an intuitive function of the engram in the hippocampal CA1, where it is proposed to index experiences stored in upstream networks as memories. Here, interpretation of the indexing function is hindered by the dual role of hippocampal cells encoding place and experience. Because the content of the engram is heavily influenced by the perception of Euclidean space, how the encoding of space contributes to formation of episodic memory remains an enigma. We have also reviewed what is known about *how* cells are recruited to an engram, first by summarizing the excitability-based neuronal allocation hypothesis and then the importance of synaptic allocation for maintaining memory identity and fidelity. While this field of research has provided much insight about how memories are linked and discriminated within overlapping engrams, the ultimate questions in the field remain unanswered. Specifically, *what* information is encoded in the engram, and *how* is the information encoded? What are the structural and functional changes achieved to encode this information? Answers to these questions may begin to emerge with new technological advances, such as widefield circuit imaging across multiple brain regions combined with innovative behavioral paradigms which allow for strict interrogation of experience and memory encoding in rodents. It is now evident more than ever that the engram is a sparse subset of neurons recruited during experience, and the experience can be recalled with reactivation of these neurons. How the complex, multimodal information representing an experience is compressed and decompressed into the engram remains one of the greatest challenges to answer in neuroscience.

## Author Contributions

OM, JL, and LC contributed to the preparation of this manuscript. All authors contributed to the article and approved the submitted version.

## Conflict of Interest

The authors declare that the research was conducted in the absence of any commercial or financial relationships that could be construed as a potential conflict of interest.

## References

[B1] Abdou K. Shehata M. Choko K. Nishizono H. Matsuo M. Muramatsu S.. (2018). Synapse-specific representation of the identity of overlapping memory engrams. Science 360, 1227–1231. 10.1126/science.aat3810 29903972

[B2] Abraham W. C. Bear M. F. (1996). Metaplasticity: the plasticity of synaptic plasticity. Trends Neurosci. 19, 126–130. 10.1016/S0166-2236(96)80018-X 8658594

[B3] Abraham W. C. Mason-Parker S. E. Bear M. F. Webb S. Tate W. P. (2001). Heterosynaptic metaplasticity in the hippocampus *in vivo*: a BCM-like modifiable threshold for LTP. Proc. Natl. Acad. Sci. U.S.A. 98, 10924–10929. 10.1073/pnas.181342098 11517323 PMC58575

[B4] Ahmed M. S. Priestley J. B. Castro A. Stefanini F. Solis Canales A. S. Balough E. M.. (2020). Hippocampal network reorganization underlies the formation of a temporal association memory. Neuron 107, 283–291.e6. 10.1016/j.neuron.2020.04.013 32392472 PMC7643350

[B5] Avaliani N. Andersson M. Runegaard A. H. Woldbye D. Kokaia M. (2016). DREADDs suppress seizure-like activity in a mouse model of pharmacoresistant epileptic brain tissue. Gene Ther. 23, 760–766. 10.1038/gt.2016.56 27416078

[B6] Ballarini F. Moncada D. Martinez M. C. Alen N. Viola H. (2009). Behavioral tagging is a general mechanism of long-term memory formation. Proc. Natl. Acad. Sci. U.S.A. 106, 14599–14604. 10.1073/pnas.0907078106 19706547 PMC2732837

[B7] Bittner K. C. Milstein A. D. Grienberger C. Romani S. Magee J. C. (2017). Behavioral time scale synaptic plasticity underlies CA1 place fields. Science 357, 1033–1036. 10.1126/science.aan3846 28883072 PMC7289271

[B8] Bliss T. V. Collingridge G. L. (1993). A synaptic model of memory: long-term potentiation in the hippocampus. Nature 361, 31–39. 10.1038/361031a0 8421494

[B9] Bliss T. V. Lomo T. (1973). Long-lasting potentiation of synaptic transmission in the dentate area of the anaesthetized rabbit following stimulation of the perforant path. J. Physiol. 232, 331–356. 10.1113/jphysiol.1973.sp010273 4727084 PMC1350458

[B10] Bostock E. Muller R. U. Kubie J. L. (1991). Experience-dependent modifications of hippocampal place cell firing. Hippocampus 1, 193–205. 10.1002/hipo.450010207 1669293

[B11] Buschler A. Manahan-Vaughan D. (2012). Brief environmental enrichment elicits metaplasticity of hippocampal synaptic potentiation *in vivo*. Front. Behav. Neurosci. 6:85. 10.3389/fnbeh.2012.00085 23248592 PMC3522088

[B12] Buzsaki G. Buhl D. L. Harris K. D. Csicsvari J. Czéh B. Morozov A. (2003). Hippocampal network patterns of activity in the mouse. Neuroscience 116, 201–211. 10.1016/S0306-4522(02)00669-3 12535953

[B13] Cai D. J. Aharoni D. Shuman T. Shobe J. Biane J. Song W.. (2016). A shared neural ensemble links distinct contextual memories encoded close in time. Nature 534, 115–118. 10.1038/nature17955 27251287 PMC5063500

[B14] Chirillo M. A. Waters M. S. Lindsey L. F. Bourne J. N. Harris K. M. (2019). Local resources of polyribosomes and SER promote synapse enlargement and spine clustering after long-term potentiation in adult rat hippocampus. Sci. Rep. 9:3861. 10.1038/s41598-019-40520-x 30846859 PMC6405867

[B15] Cohen J. D. Bolstad M. Lee A. K. (2017). Experience-dependent shaping of hippocampal CA1 intracellular activity in novel and familiar environments. Elife 6:e23040. 10.7554/eLife.23040.022 28742496 PMC5526666

[B16] Collingridge G. L. Kehl S. J. McLennan H. (1983). Excitatory amino acids in synaptic transmission in the Schaffer collateral-commissural pathway of the rat hippocampus. J. Physiol. 334, 33–46. 10.1113/jphysiol.1983.sp014478 6306230 PMC1197298

[B17] Cressant A. Muller R. U. Poucet B. (2002). Remapping of place cell firing patterns after maze rotations. Exp. Brain Res. 143, 470–479. 10.1007/s00221-002-1013-0 11914793

[B18] Curran T. Morgan J. I. (1985). Superinduction of c-fos by nerve growth factor in the presence of peripherally active benzodiazepines. Science 229, 1265–1268. 10.1126/science.4035354 4035354

[B19] De Roo M. Klauser P. Muller D. (2008). LTP promotes a selective long-term stabilization and clustering of dendritic spines. PLoS Biol. 6:e219. 10.1371/journal.pbio.0060219 18788894 PMC2531136

[B20] Denny C. A. Kheirbek M. A. Alba E. L. Tanaka K. F. Brachman R. A. Laughman K. B.. (2014). Hippocampal memory traces are differentially modulated by experience, time, and adult neurogenesis. Neuron 83, 189–201. 10.1016/j.neuron.2014.05.018 24991962 PMC4169172

[B21] Diba K. Buzsaki G. (2007). Forward and reverse hippocampal place-cell sequences during ripples. Nat. Neurosci. 10, 1241–1242. 10.1038/nn1961 17828259 PMC2039924

[B22] Disterhoft J. F. Oh M. M. (2006). Learning, aging and intrinsic neuronal plasticity. Trends Neurosci. 29, 587–599. 10.1016/j.tins.2006.08.005 16942805

[B23] Dombeck D. A. Harvey C. D. Tian L. Looger L. L. Tank D. W. (2010). Functional imaging of hippocampal place cells at cellular resolution during virtual navigation. Nat. Neurosci. 13, 1433–1440. 10.1038/nn.2648 20890294 PMC2967725

[B24] Druckmann S. Feng L. Lee B. Yook C. Zhao T. Magee J. C.. (2014). Structured synaptic connectivity between hippocampal regions. Neuron 81, 629–640. 10.1016/j.neuron.2013.11.026 24412418

[B25] Epsztein J. Brecht M. Lee A. K. (2011). Intracellular determinants of hippocampal CA1 place and silent cell activity in a novel environment. Neuron 70, 109–120. 10.1016/j.neuron.2011.03.006 21482360 PMC3221010

[B26] Fanselow M. S. (1990). Factors governing one-trial contextual conditioning. Anim. Learn. Behav. 18, 264–270. 10.3758/BF03205285

[B27] Foster D. J. Wilson M. A. (2006). Reverse replay of behavioural sequences in hippocampal place cells during the awake state. Nature 440, 680–683. 10.1038/nature04587 16474382

[B28] Frank A. C. Huang S. Zhou M. Gdalyahu A. Kastellakis G. Silva T. K.. (2018). Hotspots of dendritic spine turnover facilitate clustered spine addition and learning and memory. Nat. Commun. 9:422. 10.1038/s41467-017-02751-2 29379017 PMC5789055

[B29] Frey U. Morris R. G. (1997). Synaptic tagging and long-term potentiation. Nature 385, 533–536. 10.1038/385533a0 9020359

[B30] Frey U. Morris R. G. (1998). Weak before strong: dissociating synaptic tagging and plasticity-factor accounts of late-LTP. Neuropharmacology 37, 545–552. 10.1016/S0028-3908(98)00040-9 9704995

[B31] Fu M. Yu X. Lu J. Zuo Y. (2012). Repetitive motor learning induces coordinated formation of clustered dendritic spines *in vivo*. Nature 483, 92–95. 10.1038/nature10844 22343892 PMC3292711

[B32] Ghandour K. Ohkawa N. Fung C. C. A. Asai H. Saitoh Y. Takekawa T.. (2019). Orchestrated ensemble activities constitute a hippocampal memory engram. Nat. Commun 10, 2637. 10.1038/s41467-019-10683-2 31201332 PMC6570652

[B33] Gois Z. Tort A. B. L. (2018). Characterizing Speed Cells in the Rat Hippocampus. Cell Rep 25, 1872–1884 e4. 10.1016/j.celrep.2018.10.054 30428354

[B34] Govindarajan A. Kelleher R. J. Tonegawa S. (2006). A clustered plasticity model of long-term memory engrams. Nat. Rev. Neurosci. 7, 575–583. 10.1038/nrn1937 16791146

[B35] Guenthner C. J. Miyamichi K. Yang H. H. Heller H. C. Luo L. (2013). Permanent genetic access to transiently active neurons via TRAP: targeted recombination in active populations. Neuron 78, 773–784. 10.1016/j.neuron.2013.03.025 23764283 PMC3782391

[B36] Guzowski J. F. McNaughton B. L. Barnes C. A. Worley P. F. (1999). Environment-specific expression of the immediate-early gene Arc in hippocampal neuronal ensembles. Nat. Neurosci. 2, 1120–1124. 10.1038/16046 10570490

[B37] Hall J. Thomas K. L. Everitt B. J. (2001). Cellular imaging of zif268 expression in the hippocampus and amygdala during contextual and cued fear memory retrieval: selective activation of hippocampal CA1 neurons during the recall of contextual memories. J. Neurosci 21, 2186–2193. 10.1523/JNEUROSCI.21-06-02186.2001 11245703 PMC6762622

[B38] Han J. H. Kushner S. A. Yiu A. P. Cole C. J. Matynia A. Brown R. A. (2007). Neuronal competition and selection during memory formation. Science 316, 457–460. 10.1126/science.1139438 17446403

[B39] Han J. H. Kushner S. A. Yiu A. P. Hsiang H. L. Buch T. Waisman A.. (2009). Selective erasure of a fear memory. Science 323, 1492–1496. 10.1126/science.1164139 19286560

[B40] Harvey C. D. Svoboda K. (2007). Locally dynamic synaptic learning rules in pyramidal neuron dendrites. Nature 450, 1195–1200. 10.1038/nature06416 18097401 PMC3425382

[B41] Hebb D. O. (1949). The Organization of Behavior; a Neuropsychological Theory, A Wiley Book in Clinical Psychology. New York, NY: Wiley.

[B42] Hetherington P. A. Shapiro M. L. (1997). Hippocampal place fields are altered by the removal of single visual cues in a distance-dependent manner. Behav. Neurosci. 111, 20–34. 10.1037/0735-7044.111.1.20 9109621

[B43] Hollup S. A. Molden S. Donnett J. G. Moser M. B. Moser E. I. (2001). Accumulation of hippocampal place fields at the goal location in an annular watermaze task. J. Neurosci. 21, 1635–1644. 10.1523/JNEUROSCI.21-05-01635.2001 11222654 PMC6762966

[B44] Josselyn S. A. Shi C. Carlezon W. A. Neve R. L. Nestler E. J. Davis M. (2001). Long-term memory is facilitated by cAMP response element-binding protein overexpression in the amygdala. J. Neurosci. 21, 2404–2412. 10.1523/JNEUROSCI.21-07-02404.2001 11264314 PMC6762400

[B45] Karlsson M. P. Frank L. M. (2009). Awake replay of remote experiences in the hippocampus. Nat. Neurosci. 12, 913–918. 10.1038/nn.2344 19525943 PMC2750914

[B46] Kastellakis G. Silva A. J. Poirazi P. (2016). Linking memories across time via neuronal and dendritic overlaps in model neurons with active dendrites. Cell Rep. 17, 1491–1504. 10.1016/j.celrep.2016.10.015 27806290 PMC5149530

[B47] Kentros C. Hargreaves E. Hawkins R. D. Kandel E. R. Shapiro M. Muller R. V. (1998). Abolition of long-term stability of new hippocampal place cell maps by NMDA receptor blockade. Science 280, 2121–2126. 10.1126/science.280.5372.2121 9641919

[B48] Kumar A. Rani A. Tchigranova O. Lee W. H. Foster T. C. (2012). Influence of late-life exposure to environmental enrichment or exercise on hippocampal function and CA1 senescent physiology. Neurobiol. Aging 33, 828.e1–828.e17. 10.1016/j.neurobiolaging.2011.06.023 PMC3226902 21820213

[B49] Kuo A. G. Lee G. Disterhoft J. F. (2006). Simultaneous training on two hippocampus-dependent tasks facilitates acquisition of trace eyeblink conditioning. Learn. Mem. 13, 201–207. 10.1101/lm.98406 16585795 PMC1409830

[B50] Lashley K. S. (1929). Brain Mechanisms and Intelligence: a Quantitative Study of Injuries to the Brain, Brain Mechanisms and Intelligence: a Quantitative Study of Injuries to the Brain. Chicago, IL, US: University of Chicago Press. 10.1037/10017-000

[B51] Lee A. K. Wilson M. A. (2002). Memory of sequential experience in the hippocampus during slow wave sleep. Neuron 36, 1183–1194. 10.1016/S0896-6273(02)01096-6 12495631

[B52] Lee D. Lin B. J. Lee A. K. (2012). Hippocampal place fields emerge upon single-cell manipulation of excitability during behavior. Science 337, 849–853. 10.1126/science.1221489 22904011

[B53] Leutgeb S. Leutgeb J. K. Treves A. Moser M. B. Moser E. I. (2004). Distinct ensemble codes in hippocampal areas CA3 and CA1. Science 305, 1295–1298. 10.1126/science.1100265 15272123

[B54] Leutgeb S. Mizumori S. J. (1999). Excitotoxic septal lesions result in spatial memory deficits and altered flexibility of hippocampal single-unit representations. J. Neurosci. 19, 6661–6672. 10.1523/JNEUROSCI.19-15-06661.1999 10414995 PMC6782835

[B55] Li J. Jiang R. Y. Arendt K. L. Hsu Y. T. Zhai S. R. Chen L. (2020). Defective memory engram reactivation underlies impaired fear memory recall in Fragile X syndrome. Elife 6:e61882. 10.7554/eLife.61882.sa2 33215988 PMC7679137

[B56] Liu X. Ramirez S. Pang P. T. Puryear C. B. Govindarajan A. Deisseroth K.. (2012). Optogenetic stimulation of a hippocampal engram activates fear memory recall. Nature 484, 381–385. 10.1038/nature11028 22441246 PMC3331914

[B57] Malenka R. C. Bear M. F. (2004). LTP and LTD: an embarrassment of riches. Neuron 44, 5–21. 10.1016/j.neuron.2004.09.012 15450156

[B58] Markus E. J. Qin Y. L. Leonard B. Skaggs W. E. McNaughton B. L. Barnes C. A. (1995). Interactions between location and task affect the spatial and directional firing of hippocampal neurons. J. Neurosci. 15, 7079–7094. 10.1523/JNEUROSCI.15-11-07079.1995 7472463 PMC6578055

[B59] Martin K. C. Kosik K. S. (2002). Synaptic tagging – who's it? Nat. Rev. Neurosci. 3, 813–820. 10.1038/nrn942 12360325

[B60] Martin S. J. Grimwood P. D. Morris R. G. (2000). Synaptic plasticity and memory: an evaluation of the hypothesis. Annu. Rev. Neurosci. 23, 649–711. 10.1146/annurev.neuro.23.1.649 10845078

[B61] Masamizu Y. Tanaka Y. R. Tanaka Y. H. Hira R. Ohkubo F. Kitamura K.. (2014). Two distinct layer-specific dynamics of cortical ensembles during learning of a motor task. Nat. Neurosci. 17, 987–994. 10.1038/nn.3739 24880217

[B62] Matos M. R. Visser E. Kramvis I. van der Loo R. J. Gebuis T. Zalm R.. (2019). Memory strength gates the involvement of a CREB-dependent cortical fear engram in remote memory. Nat. Commun. 10:2315. 10.1038/s41467-019-10266-1 31127098 PMC6534583

[B63] Matsuo N. (2015). Irreplaceability of neuronal ensembles after memory allocation. Cell Rep. 11, 351–357. 10.1016/j.celrep.2015.03.042 25900079

[B64] McHugh T. J. Blum K. I. Tsien J. Z. Tonegawa S. Wilson M. A. (1996). Impaired hippocampal representation of space in CA1-specific NMDAR1 knockout mice. Cell 87, 1339–1349. 10.1016/S0092-8674(00)81828-0 8980239

[B65] Miyashita T. Kubik S. Haghighi N. Steward O. Guzowski J. F. (2009). Rapid activation of plasticity-associated gene transcription in hippocampal neurons provides a mechanism for encoding of one-trial experience. J. Neurosci. 29, 898–906. 10.1523/JNEUROSCI.4588-08.2009 19176799 PMC2749324

[B66] Moita M. A. Rosis S. Zhou Y. LeDoux J. E. Blair H. T. (2004). Putting fear in its place: remapping of hippocampal place cells during fear conditioning. J. Neurosci. 24, 7015–7023. 10.1523/JNEUROSCI.5492-03.2004 15295037 PMC6729593

[B67] Morgan J. I. Cohen D. R. Hempstead J. L. Curran T. (1987). Mapping patterns of c-fos expression in the central nervous system after seizure. Science 237, 192–197. 10.1126/science.3037702 3037702

[B68] Morris R. G. Frey U. (1997). Hippocampal synaptic plasticity: role in spatial learning or the automatic recording of attended experience? Philos. Trans. R. Soc. Lond,. B,. Biol. Sci. 352, 1489–1503. 10.1098/rstb.1997.0136 9368938 PMC1692060

[B69] Moyer J. R. Thompson L. T. Disterhoft J. F. (1996). Trace eyeblink conditioning increases CA1 excitability in a transient and learning-specific manner. J. Neurosci. 16, 5536–5546. 10.1523/JNEUROSCI.16-17-05536.1996 8757265 PMC6578886

[B70] Murakoshi H. Wang H. Yasuda R. (2011). Local, persistent activation of Rho GTPases during plasticity of single dendritic spines. Nature 472, 100–104. 10.1038/nature09823 21423166 PMC3105377

[B71] Nicoll R. A. (2017). A brief history of long-term potentiation. Neuron 93, 281–290. 10.1016/j.neuron.2016.12.015 28103477

[B72] O'Keefe J. Dostrovsky J. (1971). The hippocampus as a spatial map. Preliminary evidence from unit activity in the freely-moving rat. Brain Res. 34, 171–175. 10.1016/0006-8993(71)90358-1 5124915

[B73] O'Keefe J. Nadel L. (1978). The Hippocampus as a Cognitive Map: Oxford: Claredon Press.

[B74] Park S. Kramer E. E. Mercaldo V. Rashid A. J. Insel N. Frankland P. W.. (2016). Neuronal allocation to a hippocampal engram. Neuropsychopharmacology 41, 2987–2993. 10.1038/npp.2016.73 27187069 PMC5101572

[B75] Parsons R. G. Davis M. (2012). A metaplasticity-like mechanism supports the selection of fear memories: role of protein kinase a in the amygdala. J. Neurosci. 32, 7843–7851. 10.1523/JNEUROSCI.0939-12.2012 22674260 PMC3375025

[B76] Pfeiffer B. E. Foster D. J. (2013). Hippocampal place-cell sequences depict future paths to remembered goals. Nature 497, 74–79. 10.1038/nature12112 23594744 PMC3990408

[B77] Poirazi P. Brannon T. Mel B. W. (2003). Pyramidal neuron as two-layer neural network. Neuron 37, 989–999. 10.1016/S0896-6273(03)00149-1 12670427

[B78] Radulovic J. Kammermeier J. Spiess J. (1998). Relationship between fos production and classical fear conditioning: effects of novelty, latent inhibition, and unconditioned stimulus preexposure. J. Neurosci. 18, 7452–7461. 10.1523/JNEUROSCI.18-18-07452.1998 9736664 PMC6793227

[B79] Rashid A. J. Yan C. Mercaldo V. Hsiang H. L. Park S. Cole C. J.. (2016). Competition between engrams influences fear memory formation and recall. Science 353, 383–387. 10.1126/science.aaf0594 27463673 PMC6737336

[B80] Reijmers L. G. Perkins B. L. Matsuo N. Mayford M. (2007). Localization of a stable neural correlate of associative memory. Science 317, 1230–1233. 10.1126/science.1143839 17761885

[B81] Rickgauer J. P. Deisseroth K. Tank D. W. (2014). Simultaneous cellular-resolution optical perturbation and imaging of place cell firing fields. Nat. Neurosci. 17, 1816–1824. 10.1038/nn.3866 25402854 PMC4459599

[B82] Rolls E. T. (1999). Spatial view cells and the representation of place in the primate hippocampus. Hippocampus 9, 467–480. 10.1002/(SICI)1098-1063(1999)9:4<467::AID-HIPO13>3.0.CO;2-F 10495028

[B83] Rubin A. Geva N. Sheintuch L. Ziv Y. (2015). Hippocampal ensemble dynamics timestamp events in long-term memory. Elife 4:e12247. 10.7554/eLife.12247.016 26682652 PMC4749549

[B84] Rudy J. W. O'Reilly R. C. (1999). Contextual fear conditioning, conjunctive representations, pattern completion, and the hippocampus. Behav. Neurosci. 113, 867–880. 10.1037/0735-7044.113.5.867 10571471

[B85] Rudy J. W. O'Reilly R. C. (2001). Conjunctive representations, the hippocampus, and contextual fear conditioning. Cogn. Affect. Behav. Neurosci. 1, 66–82. 10.3758/CABN.1.1.66 12467104

[B86] Rumpel S. LeDoux J. Zador A. Malinow R. (2005). Postsynaptic receptor trafficking underlying a form of associative learning. Science 308, 83–88. 10.1126/science.1103944 15746389

[B87] Scoville W. B. Milner B. (1957). Loss of recent memory after bilateral hippocampal lesions. J. Neurol. Neurosurg. Psychiatr. 20, 11–21. 10.1136/jnnp.20.1.11 13406589 PMC497229

[B88] Sekeres M. J. Mercaldo V. Richards B. Sargin D. Mahadevan V. Woodin M. A.. (2012). Increasing CRTC1 function in the dentate gyrus during memory formation or reactivation increases memory strength without compromising memory quality. J. Neurosci. 32, 17857–17868. 10.1523/JNEUROSCI.1419-12.2012 23223304 PMC6621651

[B89] Semon R. W. Bella D. Vernon L. (1923). Mnemic Psychology. London, UK: G. Allen & Unwin.

[B90] Semon R. W. Simon L. (1921). The mneme. London, New York, NY: G. Allen & Unwin ltd.; The Macmillan company.

[B91] Shapiro M. L. Tanila H. Eichenbaum H. (1997). Cues that hippocampal place cells encode: dynamic and hierarchical representation of local and distal stimuli. Hippocampus 7, 624–642. 10.1002/(SICI)1098-1063(1997)7:6<624::AID-HIPO5>3.0.CO;2-E 9443059

[B92] Skaggs W. E. McNaughton B. L. (1996). Replay of neuronal firing sequences in rat hippocampus during sleep following spatial experience. Science 271, 1870–1873. 10.1126/science.271.5257.1870 8596957

[B93] Smith K. S. Bucci D. J. Luikart B. W. Mahler S. V. (2016). DREADDS: use and application in behavioral neuroscience. Behav. Neurosci. 130, 137–155. 10.1037/bne0000135 26913540 PMC4792665

[B94] Sorensen A. T. Cooper Y. A. Baratta M. V. Weng F. J. Zhang Y. Ramamoorthi K.. (2016). A robust activity marking system for exploring active neuronal ensembles. Elife 5:e13918. 10.7554/eLife.13918.028 27661450 PMC5035142

[B95] Swanson L. W. Mogenson G. J. (1981). Neural mechanisms for the functional coupling of autonomic, endocrine and somatomotor responses in adaptive behavior. Brain Res. 228, 1–34. 10.1016/0165-0173(81)90010-2 7023613

[B96] Takahashi N. Kitamura K. Matsuo N. Mayford M. Kano M. Matsuki N.. (2012). Locally synchronized synaptic inputs. Science 335, 353–356. 10.1126/science.1210362 22267814

[B97] Tanaka K. Z. He H. Tomar A. Niisato K. Huang A. J. Y. McHugh T. J. (2018). The hippocampal engram maps experience but not place. Science 361, 392–397. 10.1126/science.aat5397 30049878

[B98] Tanaka K. Z. McHugh T. J. (2018). The hippocampal engram as a memory index. J. Exp. Neurosci. 12:1179069518815942. 10.1177/1179069518815942 30546263 PMC6287299

[B99] Tayler K. K. Tanaka K. Z. Reijmers L. G. Wiltgen B. J. (2013). Reactivation of neural ensembles during the retrieval of recent and remote memory. Curr. Biol. 23, 99–106. 10.1016/j.cub.2012.11.019 23246402

[B100] Teyler T. J. DiScenna P. (1986). The hippocampal memory indexing theory. Behav. Neurosci. 100, 147–154. 10.1037/0735-7044.100.2.147 3008780

[B101] Teyler T. J. Rudy J. W. (2007). The hippocampal indexing theory and episodic memory: updating the index. Hippocampus 17, 1158–1169. 10.1002/hipo.20350 17696170

[B102] Titley H. K. Brunel N. Hansel C. (2017). Toward a neurocentric view of learning. Neuron 95, 19–32. 10.1016/j.neuron.2017.05.021 28683265 PMC5519140

[B103] Tonegawa S. Liu X. Ramirez S. Redondo R. (2015). Memory engram cells have come of age. Neuron 87, 918–931. 10.1016/j.neuron.2015.08.002 26335640

[B104] Ujfalussy B. B. Makara J. K. (2020). Impact of functional synapse clusters on neuronal response selectivity. Nat. Commun. 11:1413. 10.1038/s41467-020-15147-6 32179739 PMC7075899

[B105] Varela C. Weiss S. Meyer R. Halassa M. Biedenkapp J. Wilson M. A.. (2016). Tracking the time-dependent role of the hippocampus in memory recall using DREADDs. PLoS ONE 11:e0154374. 10.1371/journal.pone.0154374 27145133 PMC4856306

[B106] Wang S. H. Redondo R. L. Morris R. G. (2010). Relevance of synaptic tagging and capture to the persistence of long-term potentiation and everyday spatial memory. Proc. Natl. Acad. Sci. U.S.A. 107, 19537–19542. 10.1073/pnas.1008638107 20962282 PMC2984182

[B107] Wilson M. A. McNaughton B. L. (1993). Dynamics of the hippocampal ensemble code for space. Science 261, 1055–1058. 10.1126/science.8351520 8351520

[B108] Wilson M. A. McNaughton B. L. (1994). Reactivation of hippocampal ensemble memories during sleep. Science 265, 676–679. 10.1126/science.8036517 8036517

[B109] Winnubst J. Cheyne J. E. Niculescu D. Lohmann C. (2015). Spontaneous activity drives local synaptic plasticity *in vivo*. Neuron 87, 399–410. 10.1016/j.neuron.2015.06.029 26182421

[B110] Xu T. Yu X. Perlik A. J. Tobin W. F. Zweig J. A. Tennant K.. (2009). Rapid formation and selective stabilization of synapses for enduring motor memories. Nature 462, 915–919. 10.1038/nature08389 19946267 PMC2844762

[B111] Yang G. Pan F. Gan W. B. (2009). Stably maintained dendritic spines are associated with lifelong memories. Nature 462, 920–924. 10.1038/nature08577 19946265 PMC4724802

[B112] Yokose J. Okubo-Suzuki R. Nomoto M. Ohkawa N. Nishizono H. Suzuki A.. (2017). Overlapping memory trace indispensable for linking, but not recalling, individual memories. Science 355, 398–403. 10.1126/science.aal2690 28126819

[B113] Youngblood B. Davis C. W Ahmed R. (2010). Making memories that last a lifetime: heritable functions of self-renewing memory CD8 T cells. Int. Immunol. 22, 797–803. 10.1093/intimm/dxq437 20732857 PMC2946216

[B114] Zhang W. Linden D. J. (2003). The other side of the engram: experience-driven changes in neuronal intrinsic excitability. Nat. Rev. Neurosci. 4, 885–900. 10.1038/nrn1248 14595400

[B115] Zhou Y. Qiu L. Wang H. Chen X. (2020). Induction of activity synchronization among primed hippocampal neurons out of random dynamics is key for trace memory formation and retrieval. FASEB J. 34, 3658–3676. 10.1096/fj.201902274R 31944374 PMC7079015

[B116] Zhou Y. Won J. Karlsson M. G. Zhou M. Rogerson T. Balaji J.. (2009). CREB regulates excitability and the allocation of memory to subsets of neurons in the amygdala. Nat. Neurosci. 12, 1438–1443. 10.1038/nn.2405 19783993 PMC2783698

[B117] Ziv Y. Burns L. D. Cocker E. D. Hamel E. O. Ghosh K. K. Kitch L. J.. (2013). Long-term dynamics of CA1 hippocampal place codes. Nat. Neurosci. 16, 264–266. 10.1038/nn.3329 23396101 PMC3784308

